# Increased Hypocretin (Orexin) Plasma Level in Depression, Bipolar Disorder Patients

**DOI:** 10.3389/fpsyt.2021.676336

**Published:** 2021-05-31

**Authors:** Haimei Li, Jing Lu, Shangda Li, Bochao Huang, Gongde Shi, Tingting Mou, Yi Xu

**Affiliations:** ^1^Department of Psychiatry, The First Affiliated Hospital, Zhejiang University School of Medicine, Hangzhou, China; ^2^The Key Laboratory of Mental Disorder Management in Zhejiang Province, Hangzhou, China; ^3^Zhejiang Engineering Center for Mathematical Mental Health, Hangzhou, China; ^4^Department of Psychiatry, Brain Research Institute of Zhejiang University, Hangzhou, China

**Keywords:** plasma, hypocretin, depression, bipolar disorder, age decline

## Abstract

As hypocretin can markedly affect neurophysiological and behavioural processes in mood disorders. However, few studies have measured changes in hypocretin levels in patients with mood disorders. We estimated the hypocretin-1 plasma levels in mood disorder patients and controls (CON) using an enzyme-linked immunosorbent assay. Results: (i) The hypocretin-1 plasma level was significantly higher in major depressive disorder (MDD) patients [59.04 (35.78–80.12) pg/ml, *P* < 0.001] and bipolar disorder (BD) patients [65.50 (58.46–74.57) pg/ml, *P* < 0.001] patients than in CON [49.25 (28.51–80.40) pg/ml]. Moreover, the plasma hypocretin-1 levels in the BD group were significantly higher than those in the MDD group (*P* < 0.001). (ii). In the MDD group, patients with higher suicidal ideation had higher hypocretin-1 levels [62.09 (38.23–80.12) pg/ml] than those with lower suicidal ideation [59.63 (35.79–77.37) pg/ml), *P* = 0.032]. (iii). Plasma hypocretin-1 levels were increased in both female and male mood disorder patients compared to CON [male: MDD 60.51 (35.79–80.12) pg/ml; BD 65.40 (58.76–74.14) pg/ml; CON 45.63 (28.51–62.05) pg/ml; all *P* < 0.016; female: MDD 57.37 (34.59–80.40) pg/ml; BD 65.61 (58.46–74.57) pg/ml; CON 52.92 (38.23–78.89) pg/ml; all *P* < 0.015]. (iv). In CON, we found a significant negative correlation between plasma hypocretin-1 levels and age (rho = −0.251, *P* = 0.032), while this negative correlation was absent in the MDD and BD groups. Limitations may partly arise from the relatively small sample size and the medication history of patients participating in our research. We concluded that the clear changes found in plasma hypocretin-1 levels might be applied in the diagnosis of depression and the differential diagnosis of MDD and BD. The clear suicidal-ideation-related change found in hypocretin-1 levels in depression might be taken into account in the prevention of suicidal behaviour and further study of hypocretin-targeted therapies.

## Introduction

Depression is characterised by depressed mood and/or a loss of interest or pleasure that may adversely affect one's daily work, hobbies and general health, and it is a worldwide mental health disorder. Previously, a vicious circle was described for depression and sleep disorders (the dysregulation of hypocretin/orexin) (1–3). Hypocretin peptides (hypocretin-1 and hypocretin-2) are excitatory hypothalamic neuropeptides that play a crucial role in different physiological functions. In the past, hypocretin studies were focused on the regulation of sleep-wake function and narcolepsy (4–6), but recent studies have suggested that hypocretin plays a further role in depression, emotion processing, energy homeostasis, reward-seeking behaviour, and the regulation of endocrine functions (7–9).

It is interesting to note that the functions mentioned above are severely affected in depressed patients, and hypocretin-expressing neuron or hypocretin level changes seem to vary widely among experimental studies in rodents and humans (10). Previously, our postmortem study showed that hypocretin-1-expressing neurons were increased in depression patients compared with controls (11). Several studies have already noted the possible correlation between the hypocretin system and mood disorders. However, the results are conflicting. A recent plasma study reported lower hypocretin-1 levels in bipolar disorder (BD) patients than in controls, but no association was detected between plasma hypocretin-1 levels and any clinical symptoms, depression severity, or medication doses (12). Brundin et al. found that hypocretin levels in the cerebrospinal fluid (CSF) from suicidal patients with major depressive disorder (MDD) were reduced compared with patients suffering from adjustment disorder or dysthymia with suicide attempts (13).

In the other two studies, no significant difference was found between the mean hypocretin-1 CSF levels in patients with MDD and healthy controls, and hypocretin levels and the severity of disease were not significantly associated (14, 15). In a rodent study, the number of hypocretin-expressing neurons was significantly increased in the lateral hypothalamus of stress-induced depressed mice compared to age-matched controls (16). Feng et al. reported that adult clomipramine-treated rats with features of depression had significantly higher levels of hypothalamic hypocretin than adult controls (17). In another study, researchers found that more severe depressive behaviour was associated with reduced hypocretin expression in the hippocampus but increased hypocretin and mRNA receptor expression in the amygdala (18). These results indicated that hypocretin was likely to be involved in the pathological regulation of depression.

However, CSF hypocretin-1 concentrations are higher in the area around the hypothalamus, where hypocretin-1 is produced and released by fibres protruding into the lumen of the ventricles, as was shown in rats (19). While plasma is easier to collect in the clinic, to our knowledge, there is still a lack of studies on peripheral hypocretin-1 concentration in mood disorders. Our study aims to assess the relationship between plasma hypocretin-1 levels in mood disorder (MDD and BD) patients. We also sought to determine the relationship between hypocretin-1 levels and different aspects of mood disorders, such as suicidal ideation (SI), overall severity of depression, anxiety and sex.

## Materials and Methods

### Patients

For the plasma study, we enrolled 131 mood disorder patients, of whom 74 had MDD and 57 had BD, and 82 healthy controls. All patients were admitted to the Department of Psychiatry, the First Affiliated Hospital, Zhejiang University School, for inpatient treatment. Detailed inclusion criteria were: (1) aged 18–65 years, (2) right-handed, (3) the fulfilment of Diagnostic and Statistical Manual of Mental Disorders, fourth revision (DSM-V), and International Classification of Diseases and Related Health Problems of the World Health Organization, 10th revision (ICD-10), criteria (20) for typical or atypical BD and MDD, (4) no use of psychotic medication for the past 3 months. Exclusion criteria were: (1) history of neurological or psychiatry disorders (except MDD and BD) and MRI (Magnetic Resonance Imaging) evidence of structural brain abnormalities, (2) taking medication or drug abuse for the past 3 months, (3) patients who refuse to participate in the study. For controls, exclusion criteria were current pregnancy or psychotic disorders as well as an age of <18 years. Clinicopathological details are given in Table 1.

**Table 1:** Clinicopathological information of subjects for the plasma hypocretin-1 level study.

	**MDD (*n* = 74)**	**BD (*n* = 57)**	**CON (*n* = 82)**	**MDD and BD subgroup comparison *p*-value**	**Three-group comparison *p*-value**
Demographic variables					
Mean age (SD) (years)	27.58 (7.56)	25.04 (6.56)	27.32 (9.40)	0.057	0.22
Sex (%female)	42 (56.8)	27 (47.4)	44 (53.7)	0.288	0.562
Completed secondary education (%)	14.40 (2.70)	13.67 (3.02)	15.93 (1.62)	0.129	<0.001^*^
Suicidal ideation				0.492	
LSI (%)	41 (55.4)	36 (61.4)			
HSI (%)	33 (44.6)	21 (38.6)			
Depression severity				0.669	
LD (%)	9 (12.2)	13 (22.8)			
MD (%)	37 (50.0)	16 (28.1)			
SD (%)	27 (36.5)	28 (49.1)			
Anxiety level				0.034^*^	
LA (%)	8 (10.8)	13 (26.3)			
MA (%)	19 (25.7)	13 (26.3)			
SA (%)	47 (63.5)	27 (47.4)			

*SD, standard deviation; LSI, low suicidal ideation; HSI, high suicidal ideation; LD, low depression state; MD, moderate depression state; SD, severe depression state; SA, severe anxiety; MA, moderate anxiety; LA, low anxiety*.

* *stands for a significant statistic differences*.

### Symptom Assessment

Depression symptoms (including SI and severity) were rated using the 17-item Hamilton Depression Rating Scale (HDRS-17) before the collection of blood. The total HDRS-17 score was used to determine overall depression severity during the past few days, with scores >24 indicating a severe degree of current depression (SD), scores 17–24 representing a moderate depression state (MD), and scores ≤ 17 representing a low degree of current depression (LD). The presence of suicidality was assessed through clinical interviews and HDRS-17 item #3, which has been reported as a valid measure of SI (21). SI ratings ranged from 0 to 4, with scores ≥3 defined as a relatively high degree of SI (HSI) and scores <3 as a relatively low degree of SI (LSI).

Anxiety was measured using the Hamilton Anxiety Rating Scale (HAMA-A) (22), which is the most commonly used clinician-rated measure of anxiety in treatment studies of anxiety (23). The questionnaire consists of 14 items and reflects the level of anxiety that the participants experienced during the past few days, with scores ≥29 indicating severe anxiety (SA), scores 21–29 indicating moderate anxiety (MA), and scores below 21 indicating relatively low anxiety (LA).

### Hypocretin-1 Measurement

Following a fasting period of 8–12 h, 7 ml blood samples were extracted from the patients into tubes containing EDTA. A protease inhibitor, aprotinin, was added to the tubes (100 μl/ml blood), as recommended by the manufacturer of the enzyme-linked immunosorbent assay (ELISA) kits (Phoenix Pharmaceuticals, Inc., Belmont, CA; catalogue no. EK-003-30). The samples were centrifuged at 1,600 g at 4°C for 15 min. The plasma samples were separated and maintained at −80°C until required for analysis. Before measurements were performed, the samples were extracted according to the manufacturer's instructions using a Strata C18-E column (Phoenix Pharmaceuticals, Inc., Belmont, CA; catalogue no. S201-0253). Briefly, a solid-phase extraction (SPE) column containing 200 mg of C18 was equilibrated three times with 1 ml of Buffer B, followed by 3 ml of Buffer A. The acidified sample (mixed 1:1 with Buffer A) solution was loaded onto the pre-equilibrated C18 SPE column. The column was washed twice with 3 ml of Buffer A by a light vacuum (<10 drops/s), and the wash solution was discarded. The peptide was eluted once at a similar speed with 3 ml of Buffer B, and the eluent was collected into a polystyrene tube. The eluate was evaporated to dryness in a vacuum freeze dryer (Labconco, USA). The samples were analysed in duplicate. Hypocretin-1 levels in the plasma samples were then analysed by an ELISA, an immunoassay that determines antigen-antibody interactions, using the ELISA kits mentioned above. According to the manufacturer's instructions, the minimum detection limit of hypocretin-1 was 0.20 pg/ml.

### Statistics

The Statistical Package for the Social Sciences (SPSS) version 22.00 for Windows was used for all statistical analyses. Non-parametric tests were used because the data were not always normally distributed. Differences between 2 groups were tested using the Mann-Whitney U test. Differences among 3 groups were 1st tested with the Kruskal-Wallis (K-W) test, and if a significant difference was identified, the difference between 2 groups was checked with the Mann-Whitney *U*-test. All tests were 2-tailed, and *p* ≤ 0.05 were considered to be significant, while *p* < 0.1 and > 0.05 were considered to indicate a trend.

### Ethical Note

Our work was carried out in accordance with the Code of Ethics of the World Medical Association (Declaration of Helsinki) for experiments involving humans. This study was approved by the Research Ethics Committee of the First Affiliated Hospital, College of Medicine, Zhejiang University, and written informed consent was obtained from each participant or their family members (for severe depression study) before participation in the study.

## Results

### Plasma Hypocretin-1 Content Among All MDD and BD Patients and Controls

**Figure 1:**
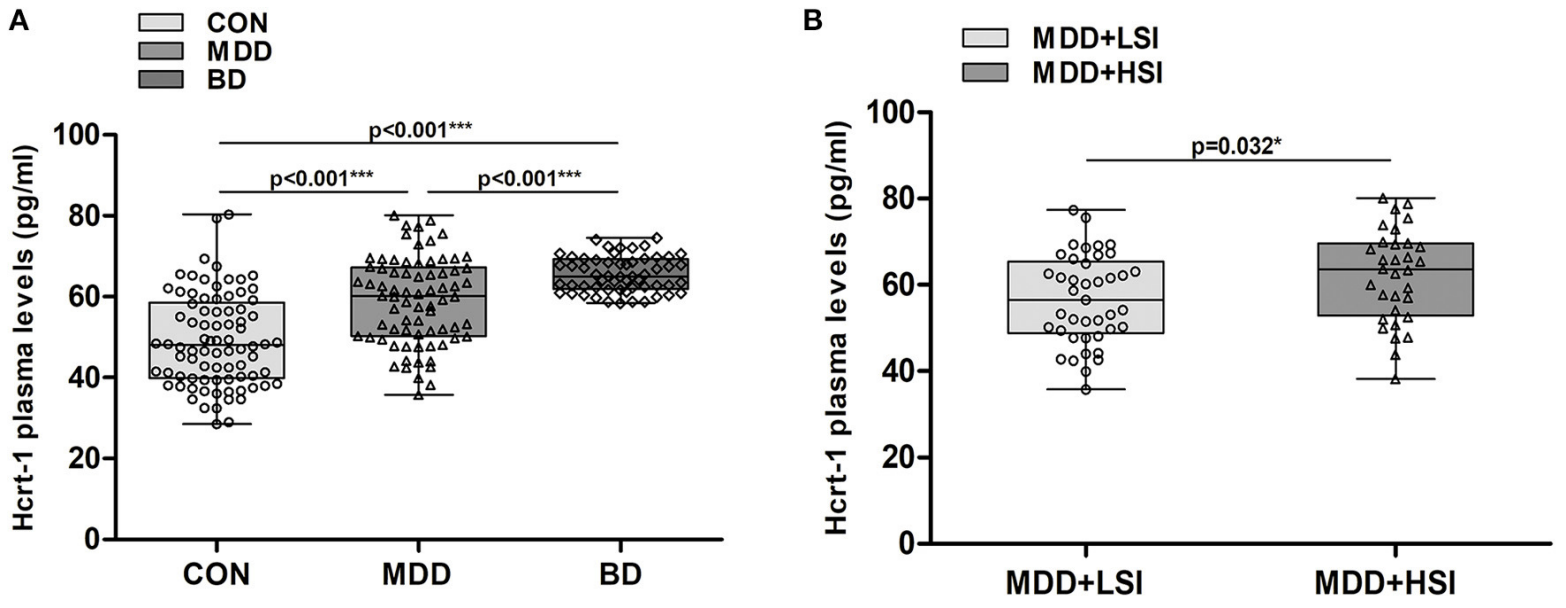
Changes in the hypocretin-1 levels in mood disorder patients. **(A)** Comparison of hypocretin-1 levels among the control (CON, *n* = 82), MDD (*n* = 74), and BD (*n* = 57) groups. Hypocretin-1 plasma levels were significantly higher in MDD and BD patients than in controls. Moreover, hypocretin-1 plasma levels were higher in BD patients than in MDD patients. **(B)** In the MDD group, patients with higher SI (*n* = 33) had higher hypocretin-1 levels than those with lower SI (*n* = 41).

MDD and BD patients and controls did not differ with respect to age, sex or episode (all *P* > 0.656). Among the three groups, we observed a significant difference (K-W: *P* < 0.001, Figure 1A), in that plasma hypocretin-1 levels were significantly higher in MDD [59.04 (35.78–80.12) pg/ml] and BD [65.50 (58.46–74.57) pg/ml] patients than in controls [49.25 (28.51–80.40) pg/ml]. Moreover, hypocretin-1 levels were higher in BD patients than in MDD patients (*P* < 0.001, Figure 1A).

### Plasma Hypocretin-1 Levels in MDD and BD Patients With Different Characteristics

In the MDD group, patients with higher SI had higher hypocretin-1 levels than those with lower SI [62.09 (38.23–80.12) pg/ml vs. 56.63 (35.79–77.37) pg/ml, *P* = 0.032, see Figure 1B], but this difference was not found in the BD group (*P* = 0.926). No difference was found in hypocretin-1 levels among individuals with different depression severities (both K-W: *P* > 0.363) or among those with different levels of anxiety (both K-W: *P* > 0.266).

### Plasma Hypocretin-1 Levels in Female and Male Groups

**Figure 2:**
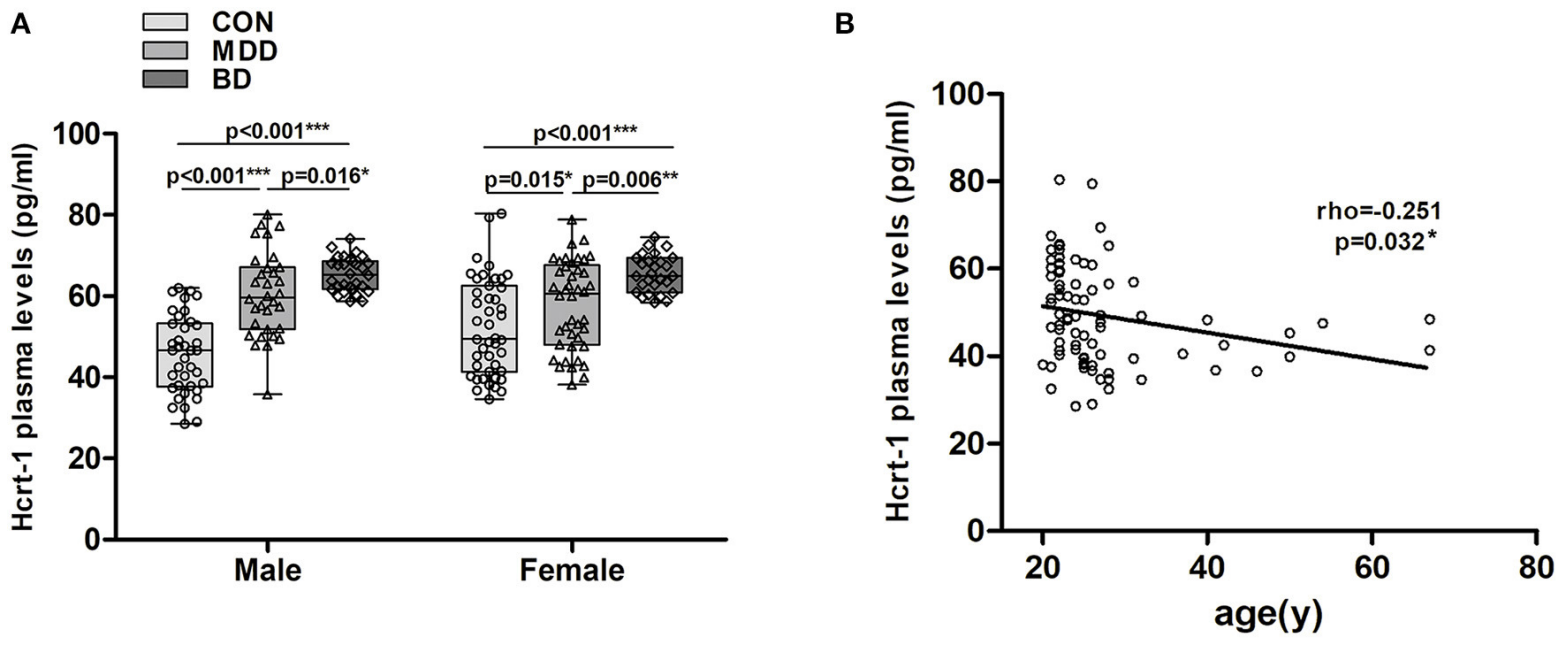
Changes in plasma hypocretin-1 levels in mood disorder patients and controls with respect to mood disorder subgroups, sex, and age. **(A)** In males, a significant increase in plasma hypocretin-1 levels was found in MDD (*n* = 32) and BD (*n* = 29) patients compared to controls (*n* = 38), and BD patients had a higher hypocretin-1 level than MDD patients. A similar difference was also observed in the female groups. **(B)** In controls, there was a significant negative correlation between plasma hypocretin-1 levels and age.

In males, a significant increase in plasma hypocretin-1 levels was found in MDD 60.51 (35.79–80.12) pg/ml) and BD [65.40 (58.76–74.14) pg/ml] patients compared to controls [45.63 (28.51–62.05) pg/ml], and BD patients had higher hypocretin-1 levels than MDD patients (all *P* < 0.016, Figure 2A). A similar difference was also observed in the female groups [MDD 57.37 (34.59–80.40) pg/ml, BD 65.61 (58.46–74.57) pg/ml, control 52.92 (38.23–78.89) pg/ml, all *P* < 0.015, Figure 2A].

### Correlation Between Plasma Hypocretin-1 Levels and Clinical Variables

In controls, we found a significant negative correlation between plasma hypocretin-1 levels and age (rho = −0.251, *P* = 0.032, Figure 2B). This correlation disappeared in the MDD (*P* = 0.471) and BD groups (*P* = 0.371). No correlation between plasma hypocretin-1 levels and any clinical variables, including secondary education year, or HAM-D and HAM-A scores, was observed.

## Discussion

In the current study, we aimed to examine plasma concentrations of hypocretin-1 and their relationship with clinically relevant psychological symptoms in mood disorder patients. We demonstrated that plasma hypocretin-1 levels were significantly increased in mood disorder patients compared with controls. Notably, the plasma hypocretin-1 levels of MDD patients were significantly higher than those of BD patients. Moreover, MDD patients with high SI showed higher plasma hypocretin-1 levels than MDD patients with low SI, while a similar difference was not found in the BD group. In controls, we found a significant negative correlation between plasma hypocretin-1 levels and age. Plasma hypocretin-1 levels were increased in both female and male MDD and BD patients compared to controls.

Our present findings indicate that plasma hypocretin-1 levels are increased in MDD and BD patients. Several studies have provided evidence suggesting that hypocretin-1 may participate in the pathophysiology of mood disorders, although the results are debatable. A recent plasma study found lower hypocrein-1 levels in BD patients than in controls, though no statistical significance was found between MDD patients and controls (12). In addition, a strong correlation was found between CSF and plasma hypocretin-1 concentrations in both patients with PTSD and healthy subjects, which suggests that plasma hypocretin-1 levels could reflect the level in CSF to some extent (24). Salomon reported higher CSF hypocretin-1 levels in depression patients than in control subjects, though the circadian amplitude was reduced in depression patients (3%) (25). However, these findings were not reproduced in another study, which found that hypocretin-1 levels in CSF were not altered in MDD patients compared to healthy controls (14). In line with our discovery, increased hypocretin neuron signalling (26) and upregulated hypocretin receptor expression in the basolateral amygdala (27) have been demonstrated in mouse depression models. On the other hand, consistent with our findings, a number of studies reported that reduced levels of hypocretin-1 have antidepressant properties. In studies of rodents, researchers showed that benzodiazepines, haloperidol and fluoxetine, which are conventional antidepressants taken by depressed patients, may inhibit hypocretin neurons and/or decrease hypocretin levels (28–30). Notably, dual hypocretin antagonist almorexant treatments suppress the physical and behavioural alterations produced by unpredictable chronic mild stress (UCMS), and almorexant administration restores stress-related dysregulation of the hypothalamic-pituitary-adrenal (HPA) axis (31).

Two prospective studies of psychiatric inpatients (including 7 mood disorder patients and 40 MDD patients) established that treatment with suvorexant (another kind of dual hypocretin antagonist) and different doses of seltorexant (a hypocretin-2 receptor antagonist) resulted in overall improvement in the quality of sleep as well as a reduction in the severity of anxiety and depression (32, 33). Interestingly, in an animal model of depression, researchers reported that exercise could produce antidepressant effects via the suppression of hypocretin and melanin-concentrating hormone in the basolateral amygdala (27). This finding suggests that hypocretin may take part in the pathopsychology of mood disorder through the hypocretinergic–monoaminergic feedback loop, which appears to be a closed circuit. Hypocretins have been found to excite monoaminergic neurons (34, 35). It is plausible that increased brain levels of hypocretins may be a result of disinhibition from defective aminergic neurons.

Moreover, our research found a difference in hypocretin levels between BD and MDD patients. Few previous studies have reported hypocretin levels in BD patients. Shoko et al. indicated a decrease in plasma hypocretin levels in BD patients compared to controls (12); however, they did not describe the protein extraction procedure for the measurements of hypocretin-1 levels. Our BD group comprised an unequal proportion of individuals with 52 bipolar II disorder (91.2%) and 5 bipolar I disorder (8.8%), and the former was characterised by hypomanic episodes and recurrent depressive episodes (36). We hypothesised that bipolar disorder II was similar to depression at certain temporary states. Our data concerning increased hypocretin levels in BD patients compared to controls were in accordance with a previous hypothesis. Moreover, a difference in hypocretin levels was found between BD and MDD patients. However, our results do provide a strong rationale for further studies on this topic.

Another finding of our study was an increased plasma hypocretin-1 levels in MDD patients with high SI compared with MDD patients with low SI. In a previous study, increased hypocretin levels were observed in the CSF in the 1st year after a suicide attempt, and the authors' previous findings showed that low hypocretin levels were related to depressive symptoms and low CRH levels in suicide attempters (37). Previously, we reported that male depressed patients who had committed suicide showed significantly increased anterior cingulate cortex hypocretin receptor-2 mRNA expression compared to male controls, but similar results were not replicated in this study. Therefore, we believe that is likely that a process involving changes in both central and peripheral hypocretin levels and other peptides plays a role in the generation of specific psychiatric symptoms, which results in significantly increased hypocretin levels in individuals with higher SI or a suicide attempt.

Notably, a significant negative correlation between plasma hypocretin-1 levels and age in controls was observed in our research, which was well-matched with that found in a study by Nicholas et al. They found that orexin neurons were reduced in the tubular hypothalamus with normal ageing in humans (38). Our study was the first to define plasma hypocretin level changes with normal ageing, and the results further indicate that plasma hypocretin levels could reflect brain hypocretin levels to some extent.

Previously, our postmortem study reported that the amount of hypocretin immunoreactivity (ir) was significantly increased in female but not in male depression patients (11), while our plasma level study showed that an increase existed in both male and female patient groups compared to controls. Further studies need to be carried out to verify this sexual dimorphic change.

Limitations may partly arise from the relatively small sample of our research. Although some of our patients had a history of taking benzodiazepines in the last 6 months (details were missed for some reasons), all controls reported no drug intake in the past 3 months. As mentioned above, medication use may reduce hypocretin levels; therefore, the plasma hypocretin-1 level in the depression group may be underestimated with respect to the effect of medication compared to controls. Notably, our controls were mostly recruited students from Zhejiang University. A significant difference in the number of individuals who had completed secondary education was observed between mood disorder patients and controls (*P* < 0.001). More studies have to be carried out to determine whether the completion of secondary education could influence hypocretin levels.

## Conclusion

Our data further indicate that hypocretin may be directly involved in the pathology of mood disorders. A deeper understanding of the biological mechanism of hypocretin in aspects of sex, SI, and anxiety should be made. The findings of our study may be of clinical value in that they suggest that hypocretin concentrations in the circulating blood, which are much more easily obtained than those in CSF, could be used as an index of peripheral hypocretin concentration in healthy subjects and a reference value in distinguishing BD patients from MDD patients.

## Data Availability Statement

The raw data supporting the conclusions of this article will be made available by the authors, without undue reservation.

## Ethics Statement

The studies involving human participants were reviewed and approved by The First Affiliated Hospital, College of Medicine, and Zhejiang University. The patients/participants provided their written informed consent to participate in this study.

## Author Contributions

YX: funding acquisition and conceptualisation. HL: conceptualisation, writing–review & editing, and project administration. JL: writing-original draft, data curation, methodology, and investigation. SL: formal analysis, resources, visualisation, and investigation. GS: resources, investigation, and methodology. BH: investigation and resources. TM: investigation and methodology. All authors contributed to the article and approved the submitted version.

## Conflict of Interest

The authors declare that the research was conducted in the absence of any commercial or financial relationships that could be construed as a potential conflict of interest.
